# Preparation and evaluation of a small-diameter vascular graft with dual anticoagulant and anticalcification functions

**DOI:** 10.1093/rb/rbag070

**Published:** 2026-04-10

**Authors:** Xiaomeng Su, Han Wang, Hongbo Chen, Linnan Ke, Qianqian Han

**Affiliations:** Institute for Medical Devices Control, National Institutes for Food and Drug Control, Beijing 102629, China; Institute for Medical Devices Control, National Institutes for Food and Drug Control, Beijing 102629, China; Institute for Medical Devices Control, National Institutes for Food and Drug Control, Beijing 102629, China; Institute for Medical Devices Control, National Institutes for Food and Drug Control, Beijing 102629, China; Institute for Medical Devices Control, National Institutes for Food and Drug Control, Beijing 102629, China

**Keywords:** vascular grafts, anticalcification, procyanidin, biomaterial

## Abstract

Cardiovascular diseases have become one of the major threats to human health. There is an urgent clinical need for small-diameter vascular grafts to achieve long-term implantation in the body. In previous studies, some progress has been made, but the prognosis is still poor. Heparin (HEP) is an anticoagulant that is widely applied in the clinical field and commonly used for anticoagulation modification of biomaterials. However, there is an important problem: HEP has almost no direct inhibitory effect on platelet adhesion, which may become one of the potential dangers after graft implantation. In recent years, it has been found that calcification is also a key factor affecting the long-term patency of small-diameter vascular grafts, which is often ignored. Therefore, in this study, based on HEP, procyanidin (PC) was introduced to modify the thermoplastic polyurethane (TPU) grafts fabricated by electrospinning to enhance the anticoagulation and anticalcification properties simultaneously. To verify the effects of drug modification on the material, the physical and chemical characterization, biocompatibility experiments and effectiveness tests of this vascular graft were conducted. The results indicated that the HEP-PC grafts could not only inhibit the excessive proliferation of human umbilical artery smooth muscle cells to some extent but also have excellent hemocompatibility. Particularly, *in vivo* experiments showed that this graft also achieved dual anticoagulation and delayed the calcification after implantation. These results suggested that HEP-PC-modified TPU might be a promising candidate for inhibiting intimal hyperplasia and anticalcification of small-diameter grafts.

## Graphical abstract

**Graphical Abstract:**
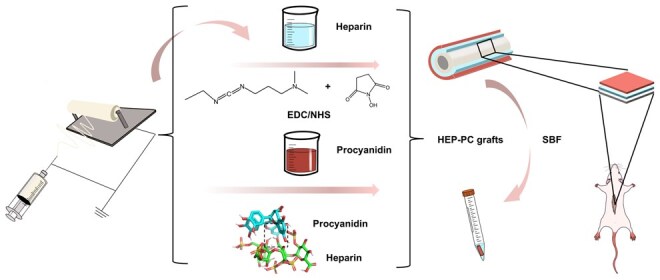

## Introduction

Currently, cardiovascular diseases (CVDs) have become one of the leading causes of health threats and mortality worldwide and the number of patients continues to rise [1]. Stenosis, occlusion or injury of blood vessels are the main pathological bases of this type of disease [2, 3]. Commonly used treatments include drug thrombolysis, percutaneous coronary angioplasty and stent intervention, with autografting being the gold standard [4, 5]. However, due to limited sources, autologous vessels cannot be successfully obtained for grafting in every case. Therefore, research on alternatives to autologous vessels is of great importance.

With 6 mm as the boundary, vascular grafts are usually classified into two categories, large-diameter and small-diameter grafts, according to their caliber. After decades of research, the properties that an ideal graft should exhibit have been recognized, including good biocompatibility, antithrombotic ability, appropriate mechanical properties. Moreover, certain endothelialization ability and compliance are also essential. Numerous studies have been conducted so far to construct vascular grafts that satisfy the above conditions. However, with the success of large-diameter grafts made of polyethylene terephthalate (PET), expanded polytetrafluoroethylene (ePTFE) and polyurethane (PU), the failure in the field of small-diameter grafts is in contrast [6]. For small-diameter vascular grafts, the most significant problem is the low rate of long-term patency, which leads to a poor prognosis for small-diameter vascular grafts. The specific causes of this phenomenon include intimal hyperplasia, thrombosis, inflammation, elastin degeneration and calcification. In previous studies, researchers were more concerned with improving the process of coagulation and thrombosis that occurs during the early stage of implantation. For instance, Norouzi and Shamloo [7] prepared a bilayer heparinized vascular graft that may reduce the probability of graft failure due to thrombosis. Cheng *et al*. [8] developed a type of extracellular matrix (ECM) scaffold and overcame the shortcomings of reduced mechanical properties and decreased biocompatibility through heparinization, which showed potent anticoagulant activity. The ability to rapidly endothelialize is also taken into consideration as a factor affecting the long-term patency rate. Several studies have been successful so far, but after the early thrombotic process, a new problem arises: calcification. The prior methods would undoubtedly reduce the risk of early embolism or inflammation; however, less consideration has been given to the calcification.

Calcification of vascular grafts refers to the deposition of minerals, such as calcium and phosphorus, on the surface or inside the material and it involves the transformation of vascular smooth muscle cells (VSMCs) to osteoblast-like cells as the starting marker [9]. Its occurrence may not only lead to graft occlusion but also to the deterioration of the mechanical properties of the material itself. In addition to graft brittleness, rupture and loss of function, shortening of material life and implantation failure are common adverse consequences. The causes of calcification are still being investigated. Some researchers believe that the calcification mechanisms of biomaterials can be broadly divided into two types: atherosclerosis-like reactions and material-specific inflammatory responses [10]. These two reactions are also interrelated or occur simultaneously. In short, calcification is an active and complex process rather than a purely passive process that occurs in relation to stimulating factors such as material properties, foreign body reactions and inflammation [11]. Hence, the application of antioxidants may inhibit graft calcification caused by oxidative stress [12]. Modulation of macrophage switching from M1 to M2 may also alleviate inflammation-induced graft calcification [13, 14].

Although some modified grafts showed excellent antithrombotic ability in the early stages of implantation, they inevitably produced varying degrees of calcification 3 months later. Compared with normal poly (ε-caprolactone) (PCL) grafts, the PCL/fibrin vascular grafts prepared by Zhao *et al*. [15] could induce vascular function reconstruction and regeneration, but there was still a small amount of calcification. Another study showed that in gelatin-coated PCL grafts, although the endothelialization process was accelerated and refined, calcification inevitably occurred after 6 months [16]. Fang *et al*. [17] also demonstrated that small-diameter grafts prepared using TPU and PCL, on which heparin (HEP) was covalently immobilized onto the surface, still showed significant calcification at 6 months, despite the continuous release of HEP and excellent anticoagulant performance. These results suggest that existing small-diameter vascular grafts cannot fully meet clinical needs and that more attention should be paid to exploring the anticalcification property of biomaterials.

In our previous study, procyanidin (PC) was utilized as a crosslinker to combine PLCL with type III recombinant humanized collagen (hCOLIII). The results showed that the prepared grafts exhibited less calcium deposition and suggested the possibility of PC for anticalcification [2]. To validate this hypothesis, a small-diameter TPU vascular graft loaded with HEP and PC was fabricated. Cytocompatibility using human umbilical artery smooth muscle cells (HUASMCs) and the human umbilical vein hybrid cell line (EA.hy926). Hemocompatibility, including hemolysis and platelet adhesion, was tested; the mechanical properties of the graft were also examined. At the same time, the *in vitro* anticalcification testing and *in vivo* implantation were performed to verify the potential of this drug-coated material as an anticalcification small-diameter vascular graft.

## Materials and methods

### Materials

TPU was obtained from Covestro (USA). Heparin sodium, procyanidin, CCK-8 kit, PC content assay kit and simulated body fluid (SBF) were purchased from Solarbio (Beijing, China).

DMEM/F-12 medium, DMEM high-glucose medium, fetal bovine serum, penicillin–streptomycin double antibody and PBS buffer were obtained from Gibco (USA).

1-(3-dimethylaminopropyl)-3-ethylcarbodiimide (EDC) and N-hydroxysuccinimide (NHS) were sourced from Adams-Beta (Shanghai, China) and Aladdin (Shanghai, China), respectively.

HUASMCs were purchased from Fenghui Biotechnology Co., Ltd. (Hunan, China). EA.hy926 was purchased from Beijing Bower Type Culture Collection (BTCC, Beijing, China). Trypsin was obtained from BioFroxx (Germany).

All animals used in the experiment were provided by the National Institutes for Food and Drug Control.

### Fabrication of the HEP-PC vascular grafts

Eight percent (8%) TPU (w/v) was dissolved in hexafluoroisopropanol (HFIP) to obtain the basic electrospinning solution. Eight percent (8%) (w/v) PEG was also dissolved in HFIP. PEG solution 1.2 mL was first discharged by a 5 mL syringe; this step was aimed at facilitating the removal of TPU grafts from the collection rod and increasing the amount of HEP attached. The solutions were injected using a syringe pump through a 20-G steel nozzle at a flow rate of 12 μL/min. The positive and negative pressures were 10 and 3 kV, respectively, at a spindle speed of 120 rpm. An iron rod (2.5 mm in diameter) was used as the collector, with a receiving distance of 17 cm.

TPU solution 3.5 mL was collected on a collection rod with PEG at the above electrospinning parameters. Later, the collection rod with the grafts was dried in a vacuum drying oven to volatilize the residual HFIP. Finally, the grafts were quickly stripped to obtain the basic TPU grafts.

EDC and NHS were used to crosslink the HEP (molar ratio of EDC/NHS = 1.6:1). Heparin sodium 0.3 g was dissolved in 30 mL MES buffer (0.05 M, pH = 5.5). TPU grafts and EDC were added to the heparin sodium solution together, followed by incubation at 37°C for 2 h. Then, the NHS was dissolved in the system to obtain a complete heparinization solution, which was incubated in a shaker at 37°C for 24 h. Unbound HEP was eliminated by washing the grafts with double-distilled water. The grafts were then dried under vacuum.

PC solution 4 mg/mL was used to coat PC. The heparinized grafts (HEP grafts) were placed in this solution and incubated in a shaker at 37°C for 36 h. Finally, the HEP-PC grafts were rinsed with double-distilled water and dried under vacuum before characterization.

### Characterization of the HEP-PC vascular grafts

#### Surface morphology characterization

The structure and morphology were observed using a scanning electron microscope (SEM; Hitachi S-4800, Japan). The grafts of each group were randomly selected for at least 150 fibers to measure and analyze the average diameter and its distribution using ImageJ software.

The pore size distribution of the grafts was determined using Hg intrusion porosimetry analysis to characterize their porosity.

#### FTIR characterization and molecular docking test

To preliminarily confirm whether PC could bind to HEP grafts, PyRx software was used for molecular docking and affinity calculations. PyMOL-2.1.0 software was used to visualize the results.

At the same time, FTIR spectra of the different materials were measured. The grafts were cut into films and completely dried, then, measured by a Nicolet 6700 FT-IR spectrometer in the range of 4000–500 cm^−1^ with 32 scans and 4 cm^−1^ resolution.

#### Mechanical properties

Axial tensile test was conducted using a universal tensile machine (H5KS, Tinius Olsen) to characterize the mechanical property of the samples. The grafts were fixed vertically on the testing machine, and stress was applied axially at a rate of 60 mm/min until the graft broke. The force required at the time of fracture was recorded, and stress–strain curves were plotted.

#### Water contact angle analysis

The water contact angle of these samples was measured using a contact angle measuring instrument (DSA100S, Krüss) to characterize their hydrophilicity or hydrophobicity. The film sample was dried and tiled. A drop of double-distilled water was deposited on the surface of the material at RT, and the contact angle was measured within 10 s. At least three samples were measured in each group, and the results were averaged.

#### The release of HEP & PC from the grafts

Heparin sodium 0.5 g was dissolved in 50 mL PBS buffer, and then, diluted to obtain a series of solutions with different concentrations. PBS buffer was regarded as a 0 mg/mL solution. Solution (2.5 mL) of each concentration was placed in a 15 mL centrifuge tube, and an equal volume of 0.005% toluidine blue solution (containing 0.2% NaCl) was added. Then, shook well for 10 min. Each tube was mixed with 5 mL n-hexane. The solution was shaken thoroughly for 15 min, and the OD value was measured at 630 nm using an ultraviolet spectrophotometer (U-3900, Hitachi) to draw the concentration-absorbance standard curve.

The HEP grafts were cut into 1 cm × 1 cm squares and placed in 2 mL of PBS buffer in each centrifuge tube. The tubes were incubated and shaken at 37°C. Different time points (0, 1, 4, 7, 14 and 28 days) were set to collect the PBS solution and add fresh PBS buffer completely. The HEP concentration released from the grafts was calculated by referring to the standard curve according to the aforementioned toluidine blue method. Finally, the HEP concentration at different time points was calculated using the following formula:


(1) $$C_{i}=\sum_{k=1}^{i}n_{k}.$$

*C_i_*: the cumulative release concentration of HEP; *n_k_*: the HEP concentration in PBS of each time interval;

The HEP-PC grafts were cut into 1 cm × 1 cm squares and put into 2 mL PBS buffer in every centrifuge tube. The tubes were incubated and shaken in a shaker at 37°C. Different time points (0, 1, 4, 7, 14 and 28 days) were set to completely collect the PBS solution and add fresh PBS buffer. The PC concentration released from the grafts was calculated using an OPC content assay kit (Solarbio, BC1350). Finally, the PC concentration at all time points was accumulated.


(2) $$C_{i}=\sum_{k=1}^{i}n_{k}.$$

*C_i_*: the cumulative release concentration of PC; *n_k_*: the PC concentration in PBS of each time interval.

### Biocompatibility evaluation

#### Cytotoxicity test

A Cell Counting Kit-8 was used to assess cytotoxicity. The grafts of each group were extracted with DMEM/F-12 medium and DMEM high-glucose medium at a rate of 120 rpm at 37°C for 24 h. The former was used to culture HUASMCs, while the latter was used for EA.hy926. Two different cells were digested and resuspended. HUASMC (100 μL) suspension was seeded into the 96-well plates at a concentration of 1 × 10^5^ cells/mL, and EA.hy926 cells were seeded in the other 96-well plates at the same cell concentration and volume. The next day, the cells were exposed to the extraction medium for 24 h, 48 h and 72 h. CCK-8 10 μL was added at predetermined intervals in each well, and the optical density (OD) value was measured at 450 nm after incubation at 37°C.


(3) $$\mathrm{Cell}\;\mathrm{Viability}\mathrm{(\%)}=\frac{A-A_{0}}{A_{0}}\times 100\%.$$

*A*: Absorbance of the test group; *A*_0_: Absorbance of the blank control group.

#### Hemolysis test

Fresh rabbit blood and 3.8% sodium citrate solution were mixed in a 1:9 ratio. Next, 5 mL 0.9% physiological saline was used to dilute 4 mL anticoagulated rabbit blood. Vascular grafts were cut into 1 cm × 1 cm squares and immersed in 0.9% physiological saline in centrifuge tubes. An equal volume of 0.9% physiological saline and distilled water was added to the other centrifuge tubes as the negative and positive control groups, respectively. All groups of centrifuge tubes were placed in a 37°C water bath for 30 min. Subsequently, 0.2 mL diluted rabbit blood was added and gently shaken. The water bath was then continued for 60 min. Finally, the samples were centrifuged at 3000 rpm for 5 min. The supernatant was used to measure the OD value at 545 nm. The hemolysis rate was calculated using the following formula:


(4) $$\mathrm{Hemolysis}\;\mathrm{Rate}\;(\%)=\frac{{OD}_{\mathrm{sample}}-{OD}_{\mathrm{negative}}}{{OD}_{\mathrm{positive}}-{OD}_{\mathrm{negative}}}\times 100\%.$$

#### Platelet adhesion test

The anticoagulated rabbit blood prepared in 2.4.2 was centrifuged twice consecutively at 1000 rpm and 2000 rpm for 10 min each, and the supernatant was used to obtain platelet-rich plasma (PRP). The vascular grafts were cut into 1 cm × 1 cm squares and washed with PBS before being placed in 24-well plates. PRP was added and incubated with the grafts at 37°C with shaking for 3 h. At the end of incubation, the platelets were rinsed with PBS to remove unadhered platelets and fixed with Gluta fixative for 12 h at 4°C, followed by gradient ethanol dehydration (50%, 60%, 70%, 80%, 90%, 100%) and air-drying.

Platelet adhesion on the surface of the material was observed using SEM, and fields were randomly selected for counting and statistical analysis.

### *In vitro* anticalcification assessment in simulated body fluid

Samples from each group were cut into 1 cm lengths, immersed in SBF and placed in a shaker at 37°C. All grafts were collected and washed with double-distilled water after 10 days.

The samples were dried and observed using an SEM. Simultaneously, they were subjected to calcium and phosphorus content measurements using energy dispersive spectroscopy (EDS) to examine the anticalcification effect of HEP-PC grafts compared to the other grafts.

### Subcutaneous implantation in rats

The vascular grafts were cut into about 0.8 cm × 0.8 cm size and sterilized using ultraviolet (UV) irradiation. Wistar rats of about 21 days of age were first randomly divided into two parts, which were respectively used to evaluate the calcification of materials in two different implantation periods. Then the materials were implanted subcutaneously on their backs. Each rat was implanted with two samples of each TPU, HEP and HEP-PC group. Ethics approval was received from the Institutional Animal Care and Use Committee (IACUC) of the National Institutes for Food and Drug Control [No. 2025 (A) 048].

The implantation period was 10 and 20 weeks, after which the rats were euthanized, and the implanted vascular materials were removed and rinsed with physiological saline. Then they were fixed with 4% paraformaldehyde for hematoxylin and eosin (H&E) and Von Kossa staining.

### Carotid artery replacement in rabbits

Nine New Zealand white rabbits weighing 3.3–3.6 kg were used in this study. The animals were randomly divided into three groups with three animals in each group for the implantation of vascular grafts. Ethics approval was received from the Institutional Animal Care and Use Committee (IACUC) of the National Institutes for Food and Drug Control [No. 2025 (B) 022].

The vascular grafts were soaked in 95% alcohol and sterilized with UV irradiation for 2 h. The rabbits were anesthetized intramuscularly (1 mL/kg) with a mixture of Zoletil 50, xylazine and physiological saline (1:1:8). Before surgery, the rabbits were intravenously injected at the ear margin with 1 mL of heparin sodium solution (10 mg/mL) for systemic heparinization. The carotid artery was isolated on the left side of the neck after shaving and disinfection of the skin. The proximal and distal ends were clamped shut with hemostatic clips; approximately 2 cm of the carotid artery was cut off, then, the graft was anastomosed end-to-end. (Supplementary data)

The hemostatic clips were removed, hemostasis was achieved by gentle pressure with gauze, and then, the incisions were sutured. Penicillin (100 000 units each) and streptomycin (50 000 units each) were given daily by intramuscular injection for 3 days after surgery. Heparin sodium solution (10 mg/mL) was injected subcutaneously at 2 mL daily, and oral, enteric-coated aspirin tablets were administered for 14 days. The patency of the implanted grafts was assessed using color Doppler ultrasound.

The samples implanted in the rabbits were removed 1 month later. Samples implanted subcutaneously in the rats were taken out at 10 and 20 weeks. These removed specimens and surrounding tissues were fixed in 4% paraformaldehyde and embedded in paraffin before sectioning. Sections were stained with H&E staining and Von Kossa staining according to standard operating procedures (SOP), and graft condition, cell morphology, infiltration and degree of calcification were also observed.

### Statistical analysis

The data were expressed as the mean ± standard deviation (SD) of three independent experiments. Data analysis was performed using GraphPad Prism version 10.1.2 software. One-way analysis of variance (one-way ANOVA) was used to analyze the difference between multiple groups. *P*-values < 0.05 were considered to be statistically significant, and the significance was marked as follows: *P < 0.05, **P < 0.01, ***P < 0.001.

## Results

### Characterization of the HEP-PC vascular grafts

#### Surface morphology characterization

**Figure 1:**
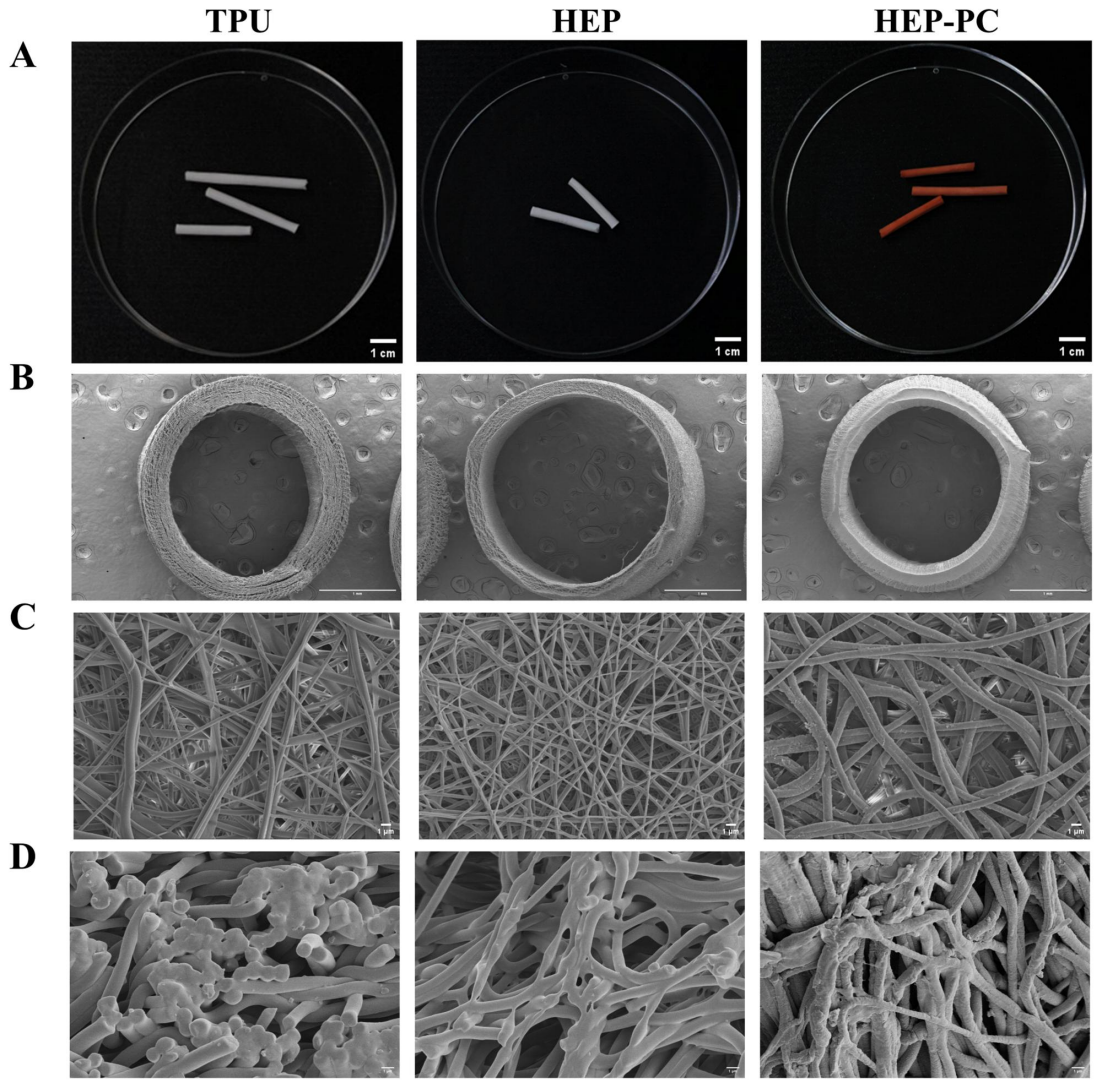
The micromorphology of vascular grafts in each group. (**A**) Direct observation. (**B**) SEM images of the grafts’ cross-section, from left to right: TPU group, HEP group, HEP-PC group, magnification 30×. (**C**) Magnification 2000×. (**D**) Magnification 5000×.

**Figure 2:**
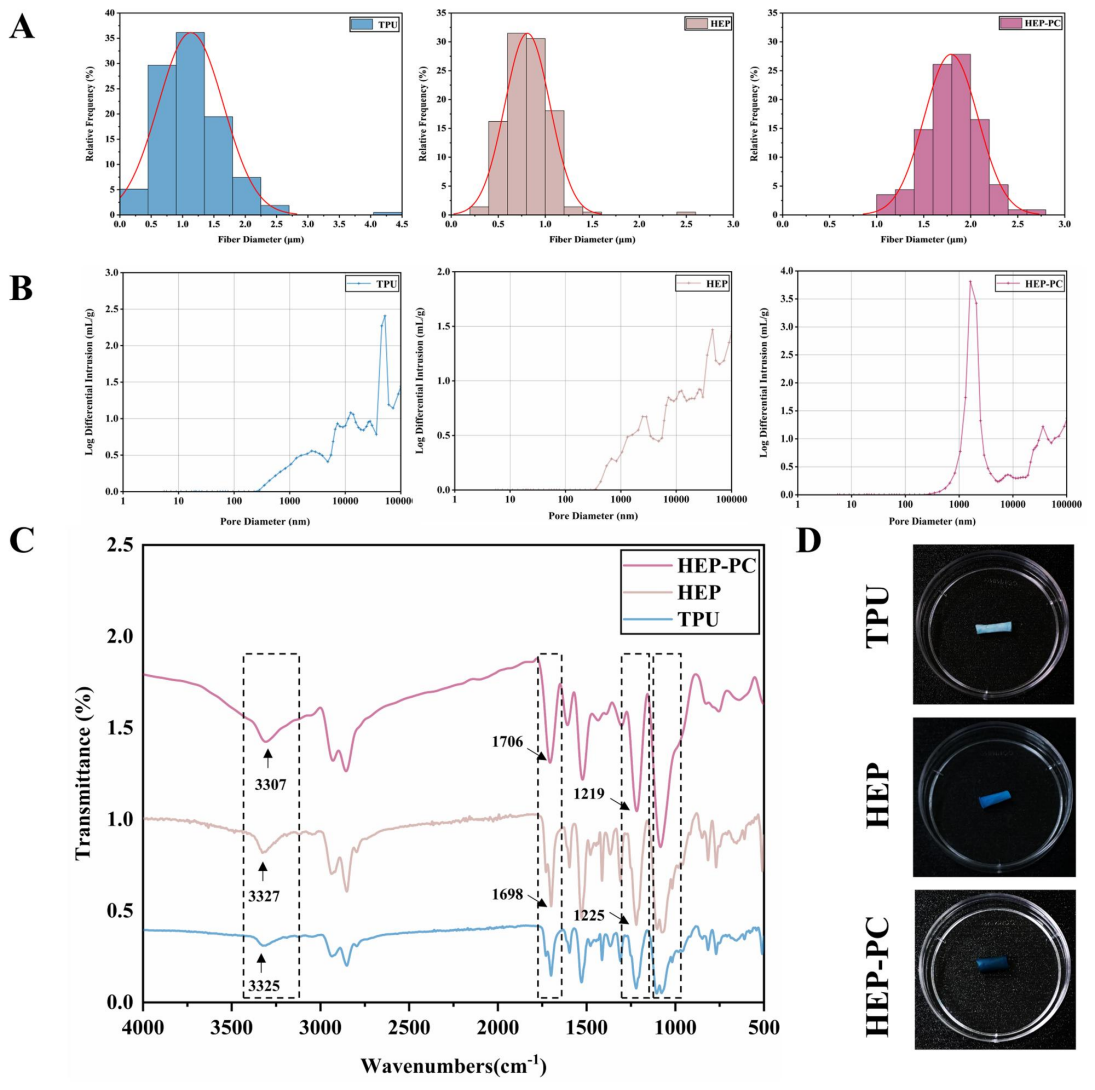
Morphology and structure characterization. (**A**) Fiber diameter distribution of the grafts, from left to right: TPU group, HEP group, HEP-PC group. (**B**) Pore size distribution of the grafts, from left to right: TPU group, HEP group, HEP-PC group. (**C**) FTIR analysis. (**D**) Toluidine blue staining test.

The appearance and micromorphology of the grafts were shown in Figure 1. Appropriate graft fiber diameter and porosity are both essential for cell adhesion and growth. As shown in Figure 2A, the fiber diameters of TPU grafts, HEP grafts and HEP-PC grafts were 1.06274 ± 0.0307 μm, 0.80465 ± 0.01009 μm and 1.81397 ± 0.00892 μm, respectively. Compared to TPU grafts, the addition of HEP slightly decreased the average fiber diameter of the HEP groups. However, after loading with PC, the diameter became larger, and their distribution became more regular. Moreover, the Hg intrusion analysis results showed that a more uniform pore size was obtained (Figure 2B), which provided more consistent spaces that can directionally guide cell proliferation and migration. From the perspective of mechanical properties, uniform and thicker fibers indicated more stable strength, making the grafts less likely to deform and rupture after being stressed.

#### FTIR characterization and molecular docking test

The FTIR results of each group were shown in Figure 2C. Characteristic range (3500–3000 cm^−1^) of hydrogen bonding. The results showed that with the loading of PC, the absorption peak in this range was red-shifted. The peak shape was broadened, and the peak intensities increased significantly. One of the characteristic peaks (1260–1220 cm^−1^) of the sulfonic group (-SO_3_) in HEP, where the peak shape was slightly broadened from sharp with a slight red shift of wave numbers.

Meanwhile, in the C=O stretching vibration region (1750–1600 cm^−1^), the peak shape of PC grafts changed and was slightly blue-shifted compared with HEP grafts. We believed that it might be due to the effect of the binding between HEP and PC on the electron cloud density. In addition, at 1150–1000 cm^−1^, an obvious change in peak shape occurred throughout the fingerprint region, and the superposition and broadening of the peak shape, as well as the increase in relative intensity, could be seen.

**Figure 3:**
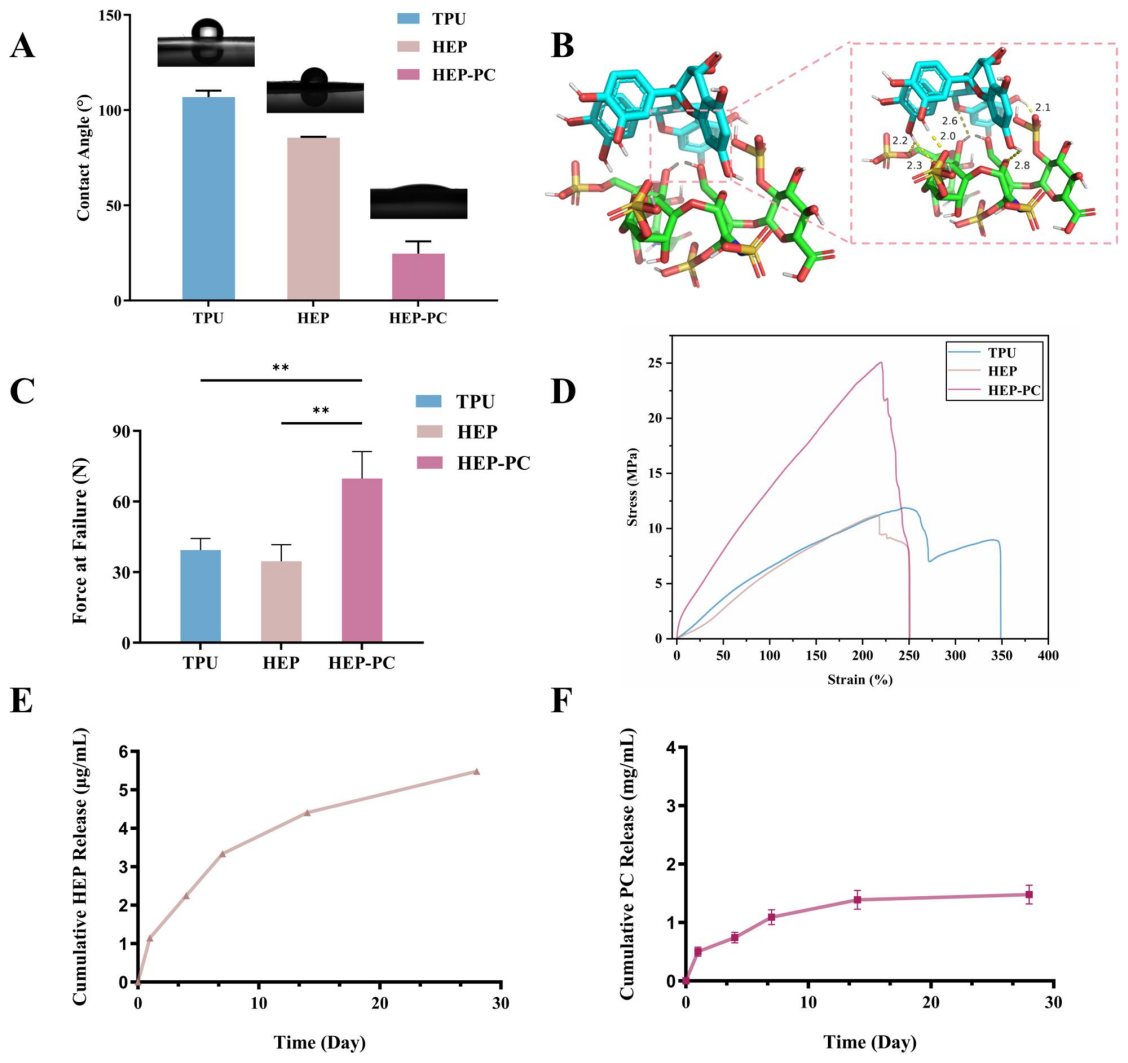
Physical and chemical characterization. (**A**) Water contact angle test (*n* = 3). (**B**) The results of molecular docking confirmed the grafting mode of HEP and PC. (**C**) Force at failure (*n* = 3). (**D**) Stress–strain curves. (**E**) Cumulative release curves of HEP (*n* = 6). (**F**) Cumulative release curves of PC (*n* = 4; ^**^P < 0.01).

These results indicated that PC and HEP produce an interaction and bind through the formation of hydrogen bonds. The molecular docking result also demonstrated the possibility of hydrogen bond formation between PC and HEP with an affinity of −4.9 kcal/mol (Figure 3B).

#### Mechanical properties

The morphology of electrospun fibers affects the mechanical strength and biocompatibility of graft scaffolds [18]. Finer fibers have good flexibility and stretchability, while coarser fibers endow the grafts with higher mechanical strength and modulus, making them less likely to break or deform under external force. We tested the tensile properties of each group of grafts (Figure 3C and D).

After PC modification, the tensile strength of the graft was enhanced. Compared with TPU grafts, the force at failure of HEP-PC grafts increased significantly, and they were less likely to break during axial stretching, which indicated that the loading of the PC significantly increased the mechanical strength of the graft.

#### Water contact angle analysis

Water contact angle is one of the important indicators to measure the hydrophilicity of biomaterials. The interaction between the material surface and platelets is also closely related to hydrophilicity. Protein adsorption on the surface of the implant, which in turn leads to platelet adhesion, is key to thrombosis [19]. A hydrophilic surface could inhibit the adhesion of plasma proteins, thereby reducing the probability of coagulation. Therefore, it is also important to regulate the hydrophilic properties of the material.

As shown in Figure 3A, the water contact angles of HEP grafts and HEP-PC grafts gradually decreased after drug modification, indicating that the surface hydrophilicity of the materials was significantly improved.

#### The release of HEP & PC from the grafts

As shown in Figure 2D, the surfaces of HEP grafts and PC grafts were dyed a deeper color with toluidine blue, demonstrating the successful grafting of HEP on the material surface. A saturated solution (4 mg/mL) was chosen for PC coating. The concentration of HEP and PC released from the material was determined cumulatively. The actual PC release was detected by the kit.

The results showed that HEP was released rapidly in the first 3 days, while the cumulative release of PC was up to 1.61 mg (Figure 3E and F). Both were released slowly from days 10 to 28. Based on the release curve, the drug exhibited rapid release in the initial stage, potentially aiding in maintaining patency for at least 1 week after graft implantation and reducing the risk of early thrombosis.

### Biocompatibility evaluation

#### Cytotoxicity test

**Figure 4:**
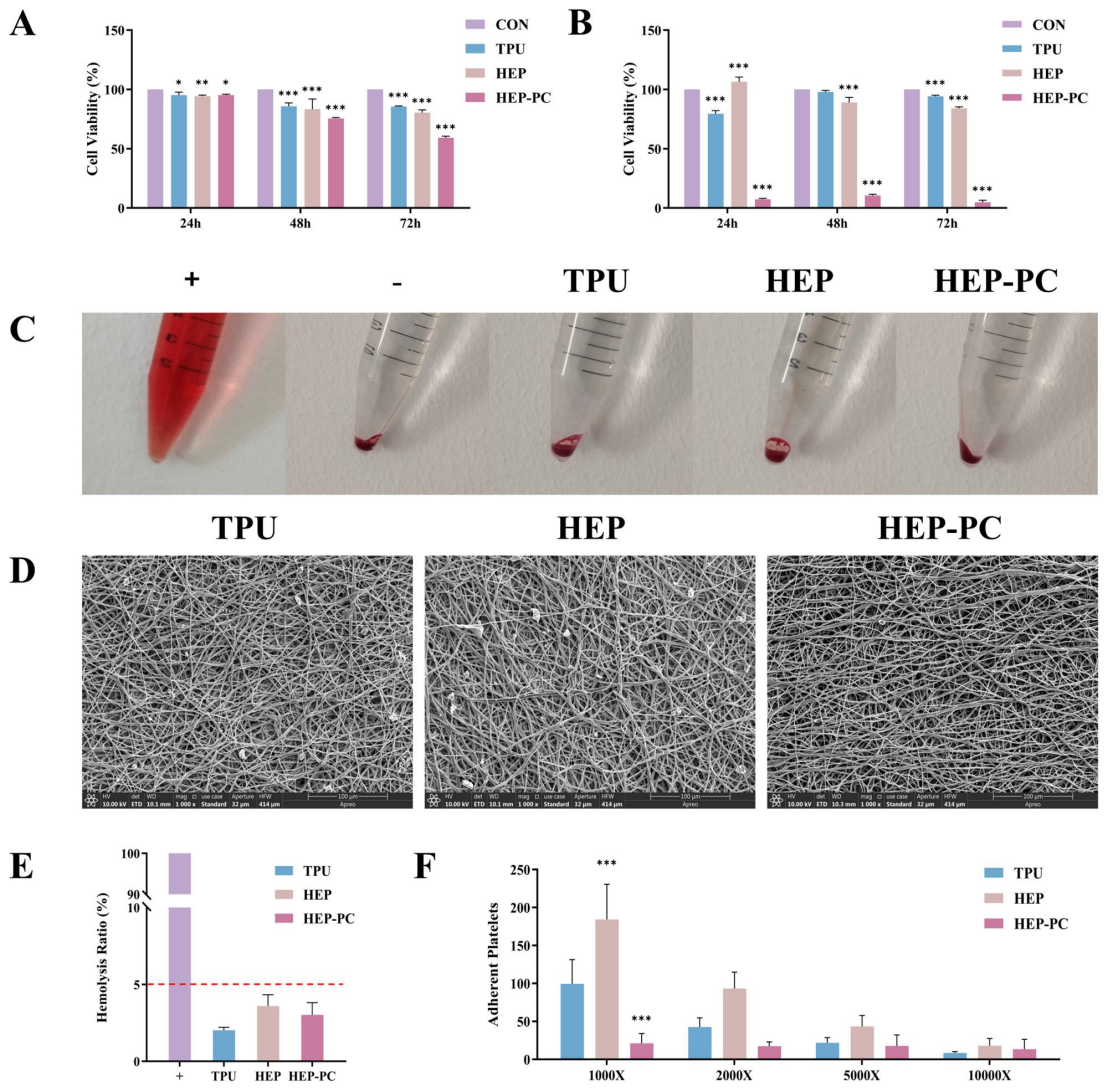
The results of the cell and blood compatibility test. (**A**) EA.hy926 cell proliferation test (*n* = 6). (**B**) HUASMCs cell proliferation test (*n* = 6). (**C**) Hemolysis test. (**D**) SEM images of platelet adhesion. (**E**) Hemolysis rate of each group (*n* = 3). (**F**) Platelet adhesion counts under random fields of view (*n* = 6; *P < 0.05, **P < 0.01, ***P < 0.001).

The results of cytotoxicity suggested that the graft extracts had a certain inhibitory effect on both types of cells (Figure 4A and B). Especially with the increase of contact time, the inhibitory effect of HEP-PC grafts on cells became more obvious. However, an excessive cell inhibition rate might not mean poor biocompatibility. Previous studies have reported its biocompatibility, and there were few reports of toxicity.

We speculated that this might be due to the limitation of the drug-loading method. According to the release curve of PC, most of the drugs were released in the first 3 days, so that the drug concentration in the system rose rapidly. Moreover, the stimulating effect of PC was also amplified by 3 days of continuous contact with cells without fluid exchange. But under *in vivo* conditions, the inhibitory effect on local cell proliferation was weakened thanks to the washout of blood flow.

Conversely, this effect seemed to be “selective.” Compared with EA.hy926 cells, the HEP-PC grafts had a stronger influence on HUASMCs, even several times as much as the former. These implied that HUASMCs might be more sensitive to PC; therefore, HEP-PC grafts might have a potential inhibitory effect on the excessive proliferation of VSMCs after implantation and might effectively inhibit intimal hyperplasia. Therefore, how to achieve a sustained and controlled release of effective drugs and to enhance the inhibitory effect on intimal hyperplasia while ensuring biocompatibility is also the next direction of research.

#### Hemolysis test

Hemolysis test results were shown in Figure 4C–E. The hemolysis rates were less than 5% in all groups, and observation indicated that the degree of hemolysis is relatively mild.

These results proved that the materials caused little damage to red blood cells, and hemolysis basically would not occur after contact with blood in the body.

#### Platelet adhesion test

The contact of biomaterials with blood is a complex process. When implanted into the body as a stranger, it would trigger coagulation and lead to the formation of thrombosis. Platelets are one of the important types of factors in the hemostasis and coagulation process of the body. One of the initial characteristics of thrombosis is the activation reaction of platelets, such as adhesion, deformation, spreading and aggregation. After vascular injury, platelets are activated, and then, undergo a transition from a discoid to a spiky shape. They provided a procoagulant surface that accelerated the conversion of prothrombin to thrombin, which promoted fibrin formation [20–22]. Accordingly, the adsorption capacity of platelets on the materials is also one of the indicators for observation. The purpose of the modification was to reduce platelet adhesion on the surface of the graft after implantation.

It could be observed that the adhesion ability of the grafts’ surfaces to platelets varied among groups. Under different magnifications, there were varying degrees of platelet adhesion and induced growth (Figure 4D). The fields of view were randomly selected, and then, platelets were counted. The statistical results showed that at lower magnification, the platelet adsorption capacity on the HEP-PC grafts was significantly different from that of pure TPU grafts. But due to the randomness of the field of view selection, there was no significant difference at higher magnification.

Overall, in the 1000× field of view, it could be observed that platelets on the surface of materials of the TPU and HEP groups showed more aggregation, accompanied by the formation of pseudopodia, which undoubtedly indicated a higher degree of platelet activation. In contrast, fewer platelets and a lower extent of activation were observed. This suggested that the loaded PC, as the outer layer of grafts, had a certain hydrophilicity, which could reduce platelet adhesion and be one of the reasons for its anticoagulation effect.

### *In vitro* anticalcification assessment in simulated body fluid

**Figure 5:**
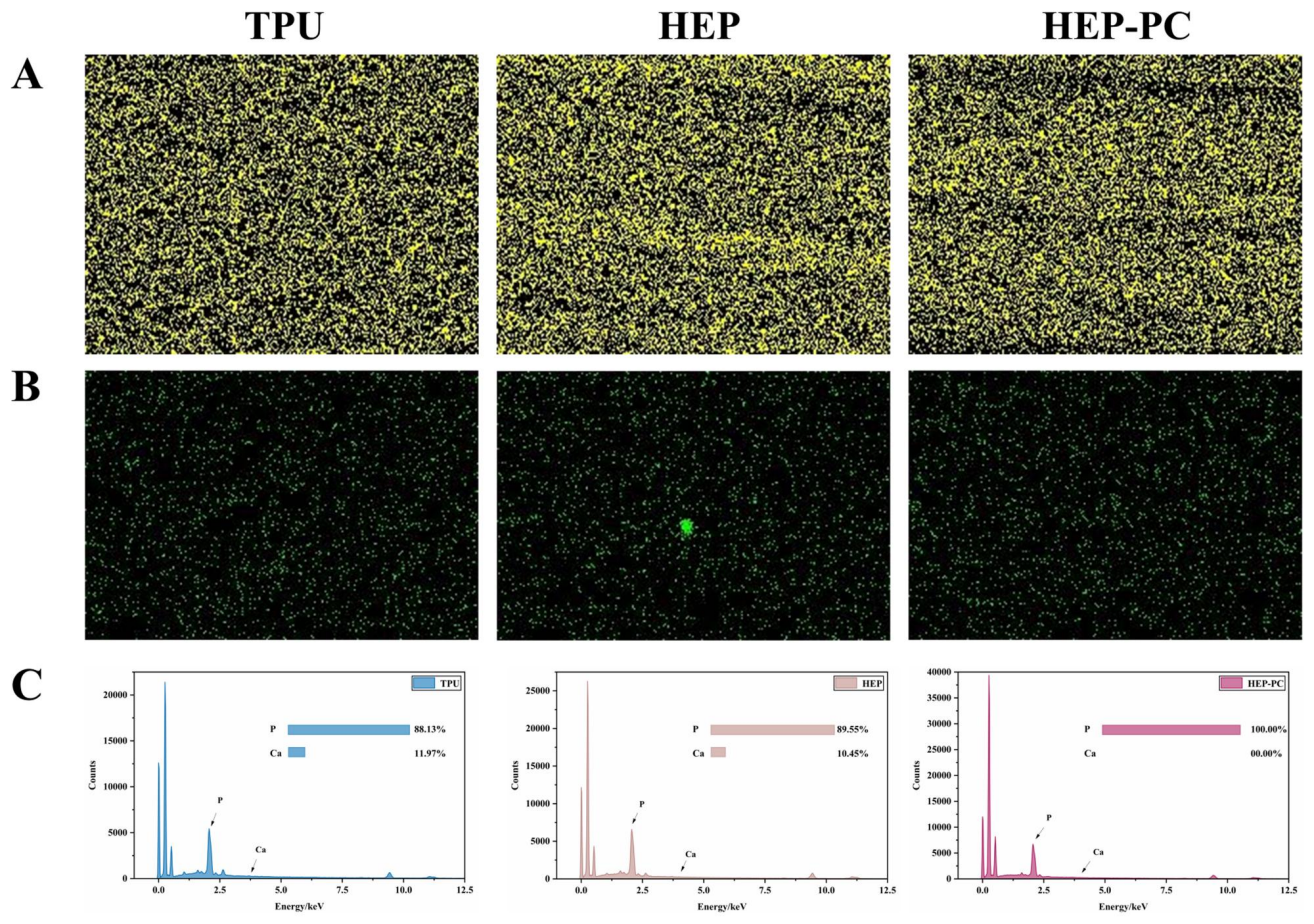
*In vitro* effectiveness experiment. (**A**) The EDS mapping images of phosphorus. (**B**) The EDS mapping images of calcium. (**C**) EDS peak spectra of the grafts in each group.

After 10 days immersed in the SBF, the grafts were dried and subjected to EDS scanning to detect the deposition of calcium and phosphorus on the surface. As shown in Figure 5, the depositions of calcium and phosphorus were basically the same, with no significant difference.

The calcium content on the surface of the materials was relatively low, while they adsorbed a large amount of phosphorus. We hypothesized that it might be due to the smooth surface of TPU grafts and their hydrophobicity, so that the adsorption of ions in inorganic solutions was inhibited. The failure of the TPU graft to induce mineralization *in vitro* by SBF suggested that the material might lack the bioactivity to induce calcium deposition in the short term and primarily exhibited physical adsorption of phosphorus. The carboxyl groups in HEP (-COOH) and the numerous hydrogen bonds in PC as a polyphenol could serve as binding sites for calcium and phosphorus. Meanwhile, after soaking and coating, the surfaces of the PC grafts became slightly rougher than the pure TPU; the grooves on the materials also provided spaces for ion deposition.

Notably, *in vitro* solution, the inhibitory effect of PC on calcium-phosphorus adsorption was not significant, a result that was contrary to expectations before. Therefore, we chose to verify it again using the *in vivo* model.

### Subcutaneous implantation in rats

The anticalcification property was initially assessed through the subcutaneous implantation model in rats. In Figure 7, it could be observed that there was a large amount of lymphocyte infiltration in all groups at 10 weeks of implantation, which was typical of inflammatory pathology. Twenty weeks after implantation, the inflammatory cells of HEP-PC grafts showed a significant reduction (Figure 8), and the degree of calcification was also mild. In some samples, calcification was not even observed. The TPU group, conversely, still showed a substantial inflammatory response accompanied by a larger area of calcification.

**Figure 6:**
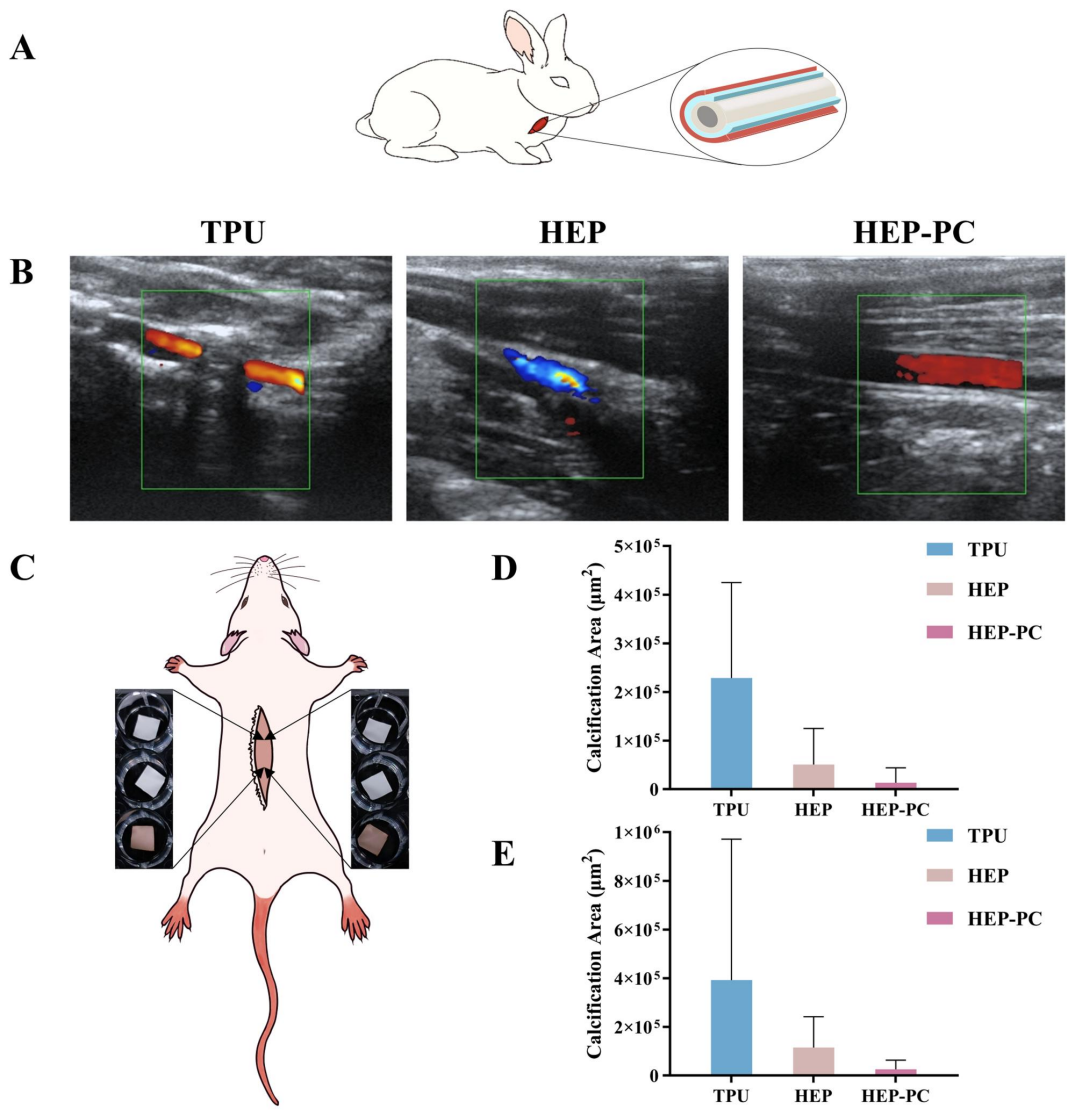
*In vivo* effectiveness experiments. (**A**) Diagram of carotid artery replacement in rabbits (*n* = 3). (**B**) Color doppler ultrasound after 1 month. (**C**) Diagram of subcutaneous implantation in rats. (**D**) Calcification area at 10 weeks (*n* = 10). (**E**) Calcification area at 20 weeks (*n* = 7).

At two time points, quantitative statistics were conducted on the calcification areas of all samples (Figure 6D and E). The overall degree of calcification in the TPU group was higher, and more samples experienced more severe calcification.

**Figure 7:**
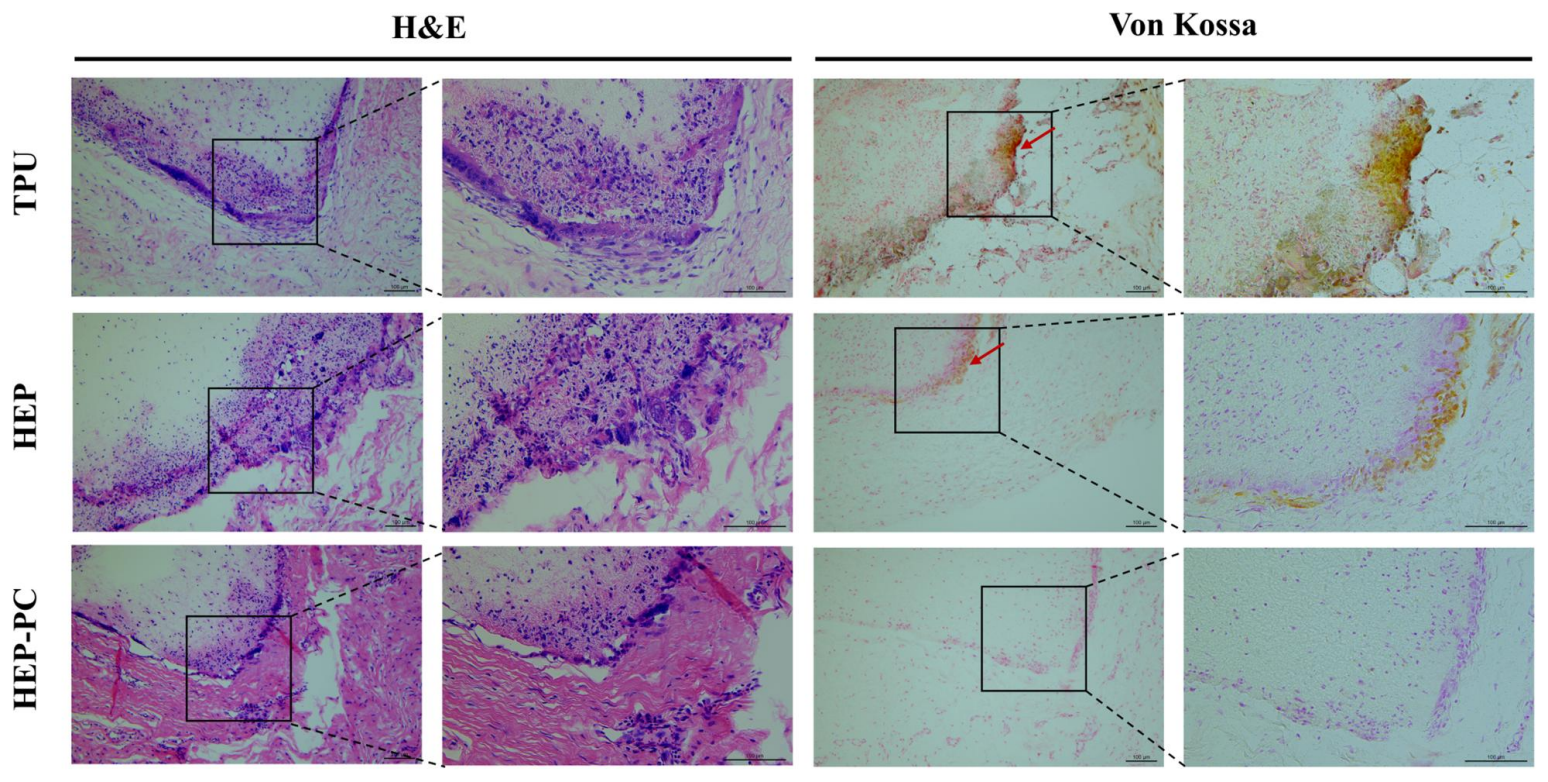
H&E staining and Von Kossa staining after 10 weeks of subcutaneous implantation. Sites of calcification were marked with arrows.

**Figure 8:**
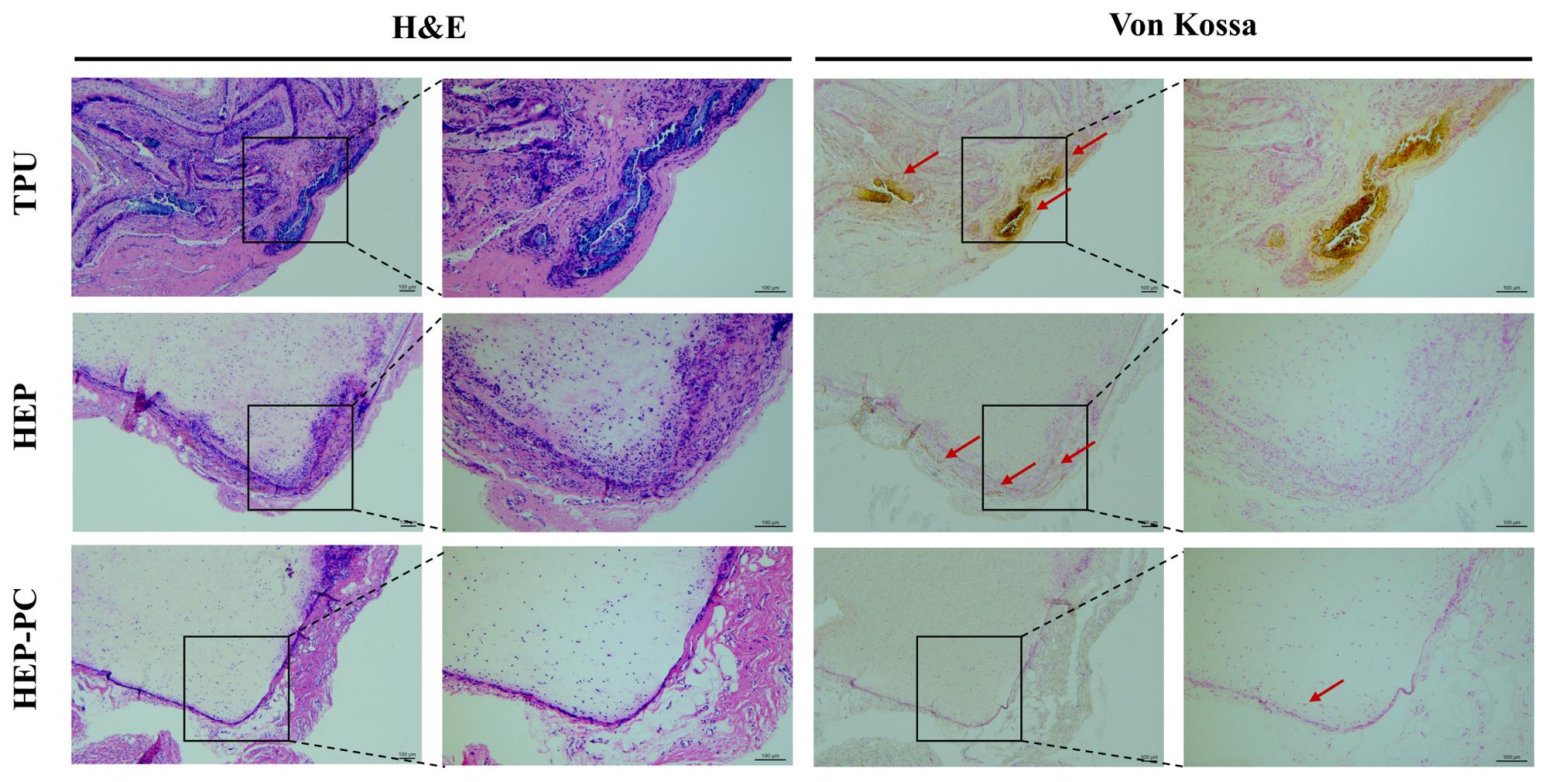
H&E staining and Von Kossa staining after 20 weeks of subcutaneous implantation. Sites of calcification were marked with arrows.

### Carotid artery replacement in rabbits

In the former test, we preliminarily verified the anticalcification effect of HEP-PC grafts by subcutaneous implantation. For further confirmation, the rabbit vascular replacement models were used for evaluation. As shown in Figure 6B, the results of color Doppler ultrasound revealed that the TPU grafts were occluded after implantation, whereas the luminal blood flow in the HEP-PC group stayed patent after 1 month. H&E staining and Von Kossa staining were performed to observe cell infiltration and calcification on the surface and inside of the grafts.

**Figure 9:**
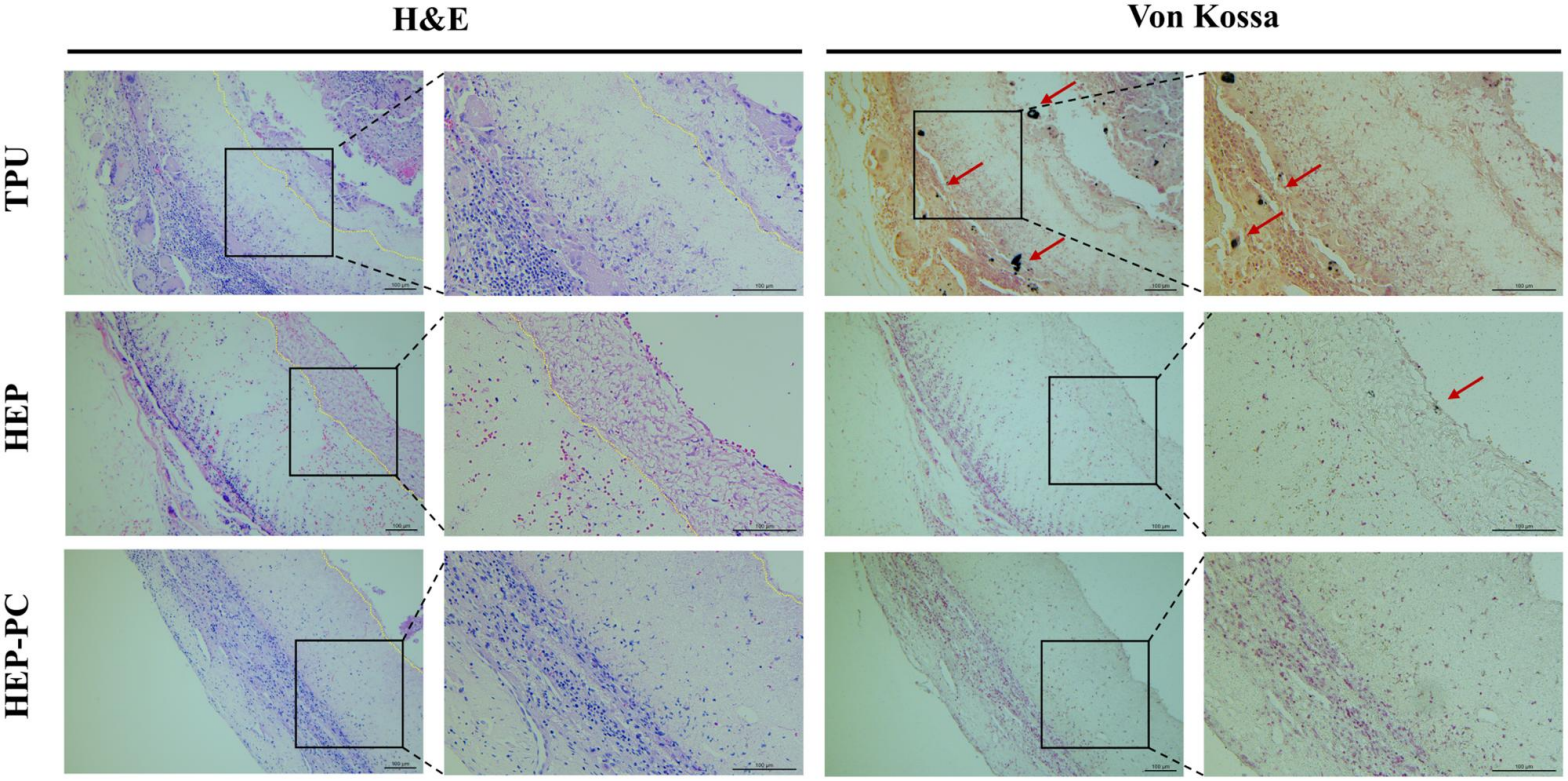
H&E staining and Von Kossa staining after 1 month of carotid artery implantation. Sites of calcification were marked with arrows. The thickened intima was separated from the graft by a yellow dashed line.

Histological analysis of each group suggested that TPU grafts and HEP grafts showed different degrees of intimal thickening (Figure 9). In contrast to the first two types, the HEP-PC grafts suggested little intimal hyperplasia. Both TPU and HEP grafts showed calcification. The former had more severe calcification, and black calcified plaques were seen inside the grafts. In the latter, only a few black calcification points were observed, and the main pathological characteristic was still intimal thickening. And almost no calcification was observed in the HEP-PC grafts.

## Discussion

The ideal small-diameter vascular grafts should possess both anticoagulation and anticalcification properties simultaneously to function at different stages after implantation and prolong the patency rate. Therefore, the aim of this study was to design a kind of graft with two components for modification, which has the synergistic effect of dual anticoagulation at the early stage of implantation and could also delay the calcification process.

Thrombosis *in vivo* is mainly driven by two systems: the coagulation factor system and the platelet system [23]. Most of the previous vascular grafts were modified based on the former and achieved an excellent antithrombotic effect. Besides their contribution to coagulation, platelets also play a crucial role in pathological clot formation by a similar mechanism in thrombosis [24]. The binding of activated platelets to fibrinogen is an important pathway in blood clot formation; unchecked activation of platelets may carry the risk of pathological thrombosis [22, 25]. Considering that both mechanisms may lead to thrombosis, the inhibition of platelet activation should be equally emphasized. We believed that it was necessary to modify both coagulation mechanisms simultaneously.

Calcification is one of the major problems after the implantation of small-diameter vascular grafts. This progressive step in vascular access increases the risk of implantation failure. Whether the grafts have excellent anticalcification properties is one of the important factors to determine if they can achieve long-term grafting.

HEP is an effective anticoagulant that is widely used in the field of medical biomaterials for postoperative anticoagulation and material modification of grafts. HEP can bind to antithrombin III (AT-III) to change its configuration and enhance its inhibitory effect on activated coagulation factors. By hindering the formation of thromboplastin, then, preventing the activation of prothrombin to thrombin, and inhibiting the conversion of fibrinogen to fibrin [26, 27], HEP can exert an anticoagulation effect. What’s more, it is beneficial for the proliferation of endothelial cells and regeneration of tissue [28]. Its inhibition of the excessive proliferation of VSMCs [29, 30] and reduction of the risk of intimal hyperplasia in grafts are also reported. Although some HEP-loaded grafts still show calcification in the middle and late periods, it has also been proven that HEP can delay the calcification of grafts to a certain extent [16], which is why we insist on HEP modification first.

Polyphenols are a class of compounds that are widely present in organisms, and in the field of cardiovascular materials, some studies have suggested that polyphenols have potential anticalcification effects [31–33]. The degenerative process leading to tissue calcification may begin with an inflammatory response triggered by xenoantigens present on the bioprosthetic tissue [34]. The anticalcification effect of polyphenols may be due to their powerful anti-inflammatory ability and capacity to mask foreign antigens. Melder *et al*. [35] used a mixed polyphenol solution to treat animal-derived valve material and showed that calcification inhibition was as high as 99.4% in polyphenol-treated samples compared to their untreated counterparts. The experiments further confirm that the protective effect of polyphenols on cardiovascular materials can be considered as a synergistic effect acting at different levels, capable of inhibiting a variety of degenerative mechanisms, including calcification.

PC is a class of natural polyphenols, which have anti-inflammatory, antioxidation and cardiovascular protection effects. At the same time, some studies have shown that PC can reduce platelet aggregation [36]. As water-soluble molecules, the addition of PC significantly improved the hydrophilicity of the grafts. The highly hydrophilic surface of the graft may reduce the formation of thrombosis by reducing the adsorption of plasma proteins and the adhesion of platelets. This type of property confers some antithrombotic ability to PC. In the area of anticalcification, previous studies using PC as a crosslinker have significantly reduced the calcification level of the obtained valves and vascular prostheses [2, 37]. In this study, the anticalcification effect of PC was verified in the graft modification and subcutaneous implantation experiment in rats. In addition, the structure of PC is rich in phenolic hydroxyl groups and is easy to form hydrogen bonds. Based on these characteristics, we decided to load the PC coating on the surface of the prepared artificial blood vessel by hydrogen bonding. By molecular docking, the possibility of a hydrogen bond between HEP and PC was confirmed.

In *in vitro* experiments, calcium attachment was slightly inhibited by PC, while the inhibitory effect on antiphosphorus adsorption was weak. From the results of *in vivo* experiments, PC has a certain effect on delaying the calcification of biomaterials. Based on histological analysis, quantification of the results of the subcutaneous implantation experiment in rats at 10 and 20 weeks showed that compared with the TPU and HEP grafts, the HEP-PC grafts had fewer calcification areas and a lower calcification rate. In the rabbit carotid artery replacement experiment, the HEP-PC grafts showed inhibition of intimal hyperplasia and calcification.

In addition, the drug delivery mode of PC also plays a key role. Previously, we attempted to use the traditional EDC/NHS method for grafting. However, PC will generate precipitated insoluble matter in the system containing EDC and cannot be grafted. We also tried to co-spin with the nozzle, but PC is insoluble in weakly polar organic solvents, while the TPU solution will expand and agglomerate when it is exposed to aqueous solvents. Therefore, PC was secondarily loaded after being modified with HEP in advance. Since HEP already exists on the surface of the graft, there is a possibility of a hydrogen bond between PC and HEP to achieve the purpose of successful loading. However, the disadvantage of this method is that the loaded PC content is less, and the PC on the graft surface will be gradually metabolized with the blood flow, which will gradually weaken the anticalcification effect in the later stages. In response to this phenomenon, replacing other solvents such as DMSO or THF is also a feasible strategy; however, the toxicity of these solvents and their potential irritation to cells must be considered.

There are limitations to our study. First of all, given the limited number of animals used as samples within *in vivo* experiments and the fact that there are still some differences between animal models and humans, the results are not fully generalizable to humans. Therefore, it is necessary to further expand the sample size and species diversity. Second, only one preliminary exploration of the drug-loading method was conducted, but its loading amount was limited, which might disrupt the evaluation of the anticalcification ability of PC. Incorporating PC during the preparation of graft materials or introducing other molecules to achieve gradual drug release represents a promising approach for achieving controlled release in the future. Finally, the *in vivo* effectiveness experiment in this study only lasted for 1 month due to the limitation of the experimenter’s surgical level, so long-term studies are urgently needed.

## Conclusion

Here, a small-diameter TPU vascular graft loaded with HEP and PC was prepared. The HEP-PC grafts were fabricated by electrospinning and loaded with HEP and PC via surface heparinization as well as immersion. Grafts fabricated using this method not only had good mechanical strength and compliance, but at the same time, the *in vivo* evaluation showed that the vascular access remained patent 1 month after implantation. With the intima thickening caused by the over-proliferation of VSMCs and platelet adhesion being inhibited, it was proven to have good antithrombotic effects, among others. The histopathological results showed that compared to the blank group (TPU grafts) and control group (HEP grafts), the HEP-PC grafts showed calcification to a lesser extent, demonstrating the anticalcification potential of PC. The results of this study may provide new ideas for the research and application of PC in the field of vascular graft materials.

## Supplementary Material

rbag070_Supplementary_Data
