# Supplementary figures and images for: Preparation and evaluation of a small-diameter vascular graft with dual anticoagulant and anticalcification functions
Source: Regen Biomater. 2026 Apr 10;13:rbag070. doi: 10.1093/rb/rbag070 (PMC13176455; doi:10.1093/rb/rbag070)

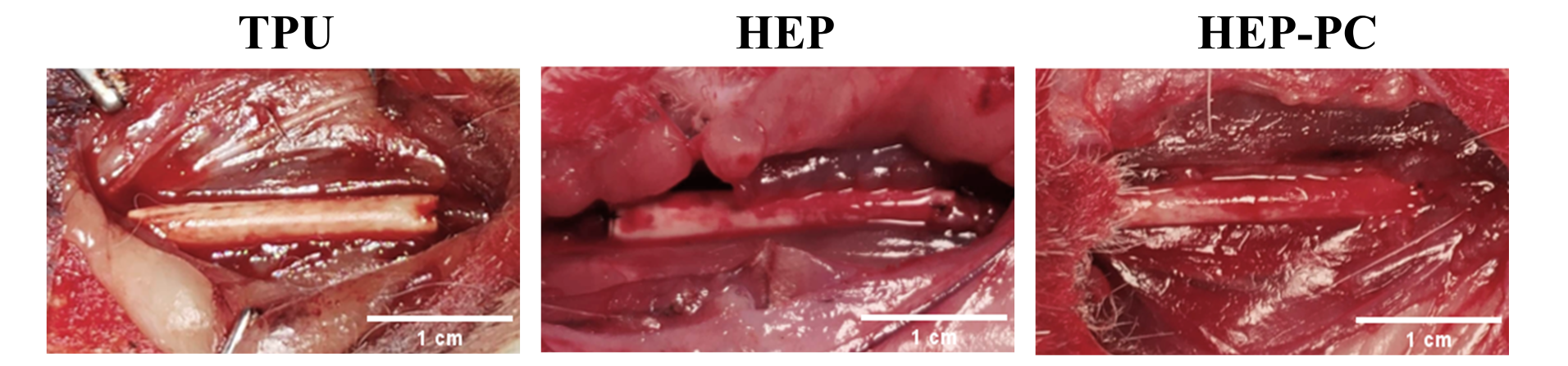

Supplement: rbag070_Supplementary_Data [file rbag070_supplementary_data.zip › Carotid artery replacement in rabbits.tif]
